# Cohort Differences in Social Participation in the National Social Life, Health, and Aging Project

**DOI:** 10.1093/geroni/igaa057.2113

**Published:** 2020-12-16

**Authors:** Linda Waite, Rebeccah Duvoisin, Ashwin Kotwal

**Affiliations:** 1 University of Chicago, Chicago, Illinois, United States; 2 NORC at the University of Chicago, Chicago, Illinois, United States; 3 University of California, San Francisco, San Francisco, California, United States

## Abstract

Has American society become more socially disconnected as Robert Putnam argues in Bowling Alone? Claude Fischer disputes this contention with evidence that Americans remain about as connected to friends and family as in the past. We address this debate with data for older adults from the National Social Life, Health and Aging Study, collected in 2005, 2010, and 2015. We compare social participation as reported at ages 57 to 68 for members of the Silent Generation cohort vs the Baby Boom cohort. We find that the gender gap in social participation evident for the Silent Generation does not exist at all for younger Baby Boomers, only appearing after age 62. These same cohort differences appear for participation in religious services and organized groups. This suggest that the gendered separation of social roles that characterized older generations is becoming less pronounced, with implications for social support and social isolation.

